# Macroprolactinoma with secondary resistance to dopamine agonists: a case report and review of the literature 

**DOI:** 10.1186/s13256-023-03820-5

**Published:** 2023-03-17

**Authors:** Eng-Loon Tng, Ada Ee Der Teo, Aye Thida Aung

**Affiliations:** 1 Department of Medicine, Ng Teng Fong General Hospital, 1 Jurong East Street 21, Tower A Level 8, Singapore, 609606 Singapore; 2 Department of Medicine, Division of Endocrinology, National University Health System, 1E Kent Ridge Road, NUHS Tower Block Level 10, Singapore, 119228 Singapore; 3 Ministry of Health Holdings, 1 Maritime Square, #11-25 HarbourFront Centre, Singapore, 099253 Singapore

**Keywords:** Bromocriptine, Cabergoline, Case report, Dopamine agonists, Macroprolactinoma, Pergolide, Prolactin-secreting adenoma, Quinagolide, Secondary resistance

## Abstract

**Background:**

Resistance to dopamine agonists is not uncommonly seen in prolactinomas. However, development of resistance to dopamine agonists after an initial period of robust treatment response is rare, and only 39 cases have been reported in the past four decades. We describe a Chinese man with this rare condition and explored the postulated mechanisms that may explain this phenomenon. We compiled similar cases that were previously reported and compared their etiology, progress, and response to treatment. On the basis of these cases, we derived a list of differential diagnoses to consider in patients with secondary resistance to dopamine agonists.

**Case presentation:**

A 63-year-old Chinese man presented with blurred vision and was subsequently diagnosed with a macroprolactinoma. He had initial response to cabergoline but developed secondary resistance to it after 5 years. The prolactinoma continued to grow, and his serum prolactin remained markedly elevated despite adherence to escalating dosages of cabergoline up to 6 mg/week. The patient finally underwent transsphenoidal surgery and was found to have a sparsely granulated lactotroph tumor with Ki-67 index of 5%. Postoperatively, there was improvement in his serum prolactin level, although he still required treatment with cabergoline at 6 mg/week.

**Conclusions:**

Surgery can facilitate disease control in patients with prolactinomas that develop secondary resistance to dopamine agonists. Malignant prolactinoma is an important differential diagnosis in this group of patients, especially when serum prolactin remains markedly elevated despite resolution or stability of the primary pituitary lesion, suggesting a metastatic source of prolactin secretion.

## Background

Prolactinomas account for 30–60% of pituitary tumors [1, 2] and occur in 44.4–62.7 per 100,000 patients in cross-sectional studies [2, 3]. Dopamine agonists (DA) are the drugs of choice to treat prolactinomas. DA normalize prolactin (PRL) levels in more than 90% of people with microprolactinomas and in 77% of people with macroprolactinomas, and reduce tumor size in up to 89% of cases [4]. However, up to 10% of patients with microprolactinomas and less than 20% of patients with macroprolactinomas may not achieve normoprolactinemia or reduction in tumor size with DA therapy [5]. About 25% of people with prolactinomas are resistant to bromocriptine (BRC) and 10–15% are resistant to cabergoline (CAB) [6]. Most cases of DA resistance are apparent soon after starting therapy, and these patients are considered to have primary DA resistance. Rarely, patients develop hyperprolactinemia and/or tumor enlargement after an initial period of remarkable response, and these patients are considered to have secondary DA resistance. We describe a Chinese man with macroprolactinoma who developed secondary DA resistance and explore the possible mechanisms and clinical implications of this phenomenon. Our case report is the first to compile previous case reports on prolactinomas with secondary DA resistance to provide a comparison on their etiology, progress, and response to treatment. On the basis of these case reports, we derived a list of differential diagnoses to consider in this group of patients.

## Methods

We searched PubMed for articles published using the terms “aggressive prolactinoma,” “bromocriptine,” “cabergoline,” “CV 205-502,” “dopamine agonist,” “dopamine agonist resistance,” “Ki-67,” “macroprolactinoma,” “malignant prolactinoma,” “microprolactinoma,” “pergolide,” “prolactinoma,” “prolactin-secreting adenoma,” “quinagolide,” and “secondary resistance.” We summarized available articles describing secondary DA resistance and contacted authors for clarification when there were inadequate or ambiguous data.

## Case presentation

**Fig. 1:**
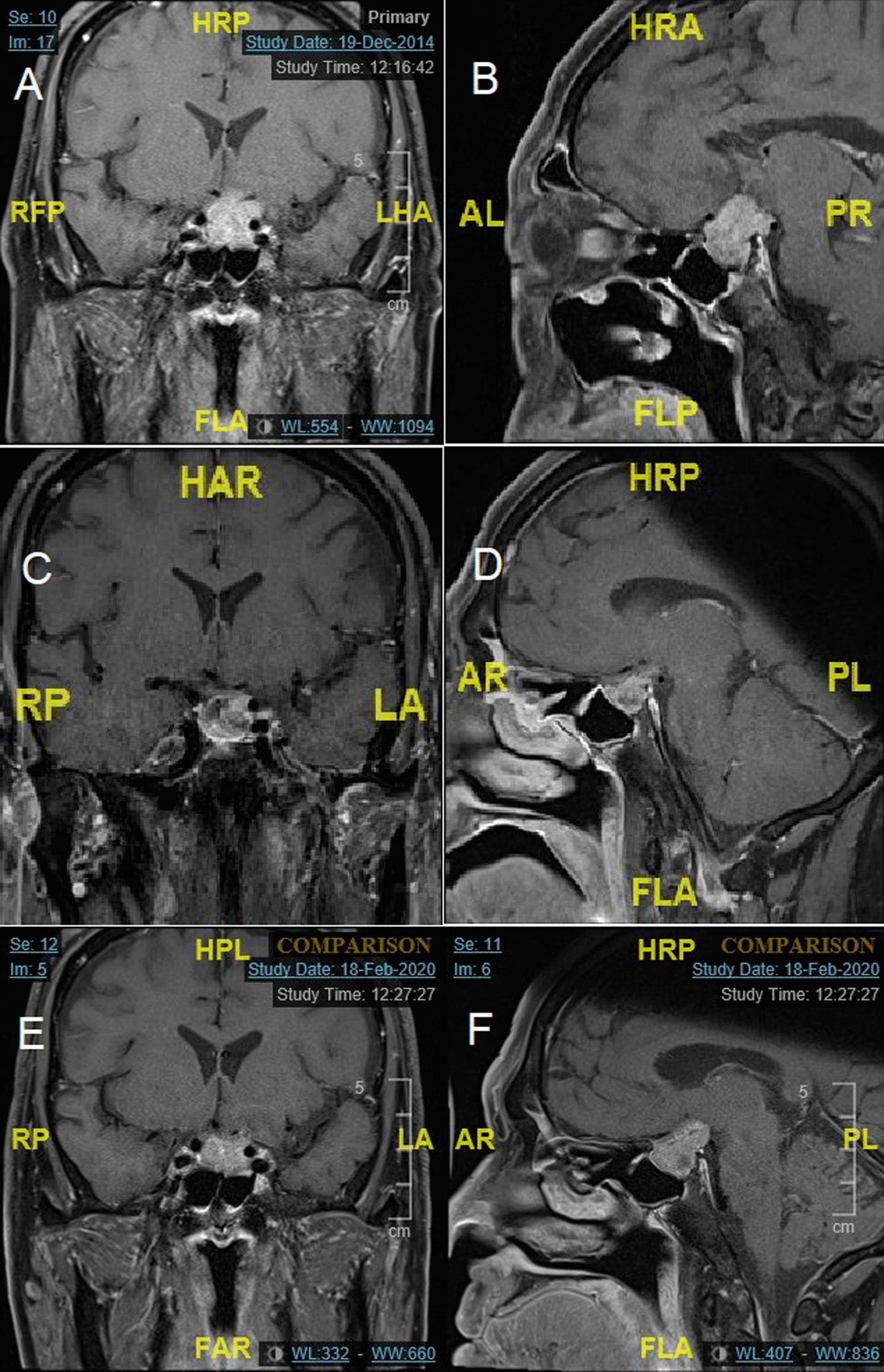
MRIs. **A**, **B** December 2014, **C**, **D** June 2015, **E**, **F** February 2020

**Fig. 2:**
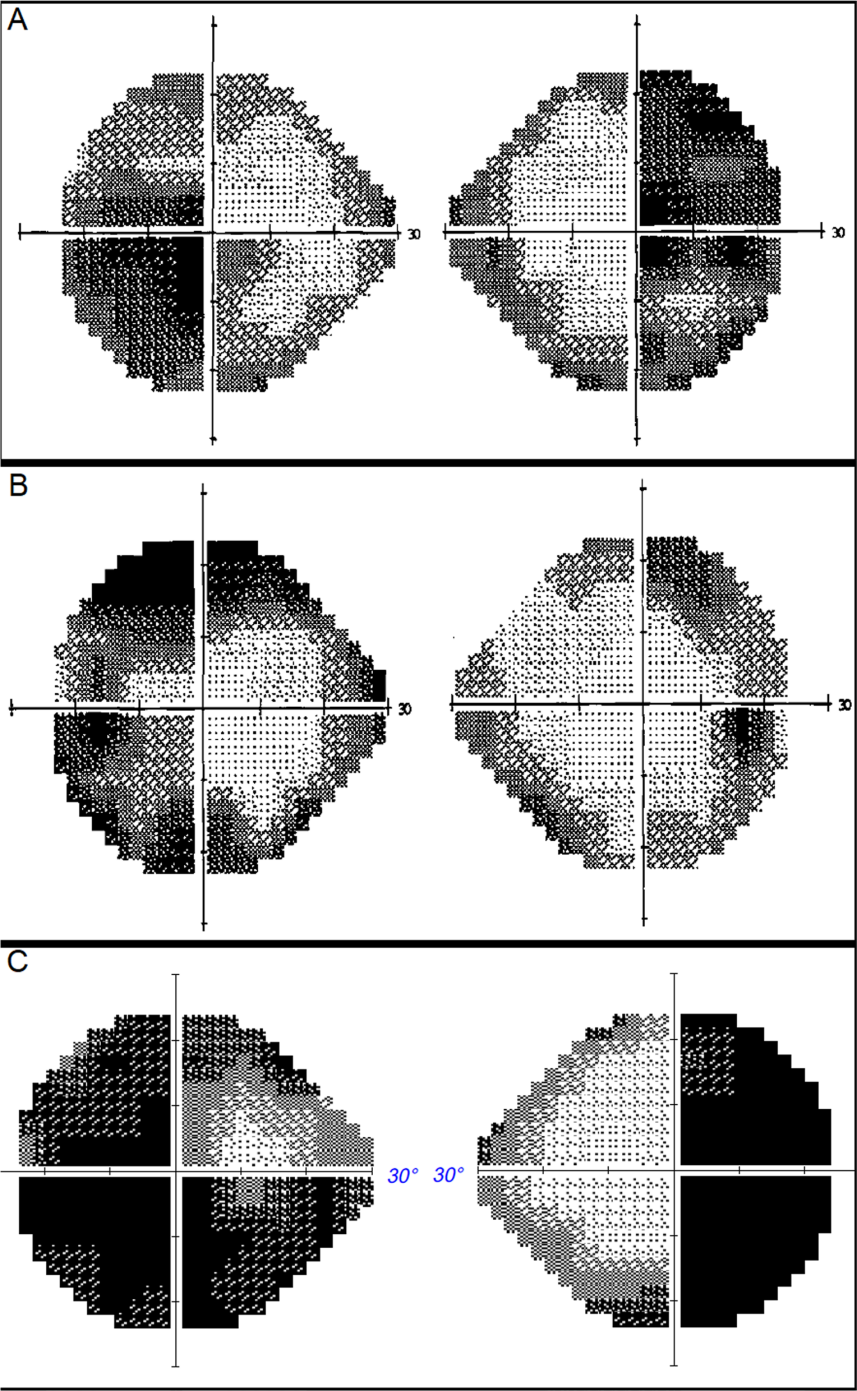
Perimetry **A** December 2014, **B** June 2015, **C** December 2019

A 63-year-old Chinese man presented in November 2014 with gradual blurring of vision in his left eye for 3 months. His past medical history included type 2 diabetes mellitus, hypertension, and hyperlipidemia. He has no family history of pituitary or endocrine disorders. He was found to have bitemporal hemianopia and magnetic resonance imaging (MRI) showed a 2.4 × 2.0 × 2.0 cm (craniocaudal, transverse, and anteroposterior dimensions) lesion in the pituitary fossa with suprasellar extension and encasement of his left cavernous sinus. The MRI and perimetry in December 2014 are shown in Figs. 1A and B, and 2A respectively.

He reported no headache, diplopia, lethargy, postural dizziness, cold or heat intolerance, galactorrhea, or reduced frequency in shaving. He was not Cushingoid or acromegalic, and was clinically euthyroid. There was no postural hypotension. He had mild gynecomastia bilaterally and no galactorrhea. His blood tests showed PRL > 470 ug/L (reference: 4.0–15.2), free thyroxine 10.7 pmol/L (reference: 11.8–24.6), thyroid stimulating hormone 0.564 mU/L (reference: 0.270–4.200), early morning total testosterone 5.16 nmol/L (reference: 9.90–27.80), follicle stimulating hormone 4.8 IU/L (reference: 1.5–12.4), luteinizing hormone 4.1 IU/L (reference: 1.7–8.6), early morning cortisol 190 nmol/L (reference 171–536), adrenocorticotropic hormone 8.6 pmol/L (reference: 1.6–13.9), and insulin-like growth factor 1 153 ug/L (reference: 75–212). His peak cortisol with the Cosyntropin test was 667 nmol/L. He was diagnosed with a macroprolactinoma with hypogonadotropic hypogonadism and central hypothyroidism.

CAB 1 mg/week and levothyroxine 25 mcg daily was started in December 2014 with good biochemical and radiological response of the macroprolactinoma. There was improvement in his visual field defects. The MRI and perimetry in June 2015 are shown in Figs. 1C and D, and 2B respectively. He defaulted treatment after February 2017 and the macroprolactinoma had expanded in size by the time he returned to the endocrinology clinic in December 2019. His left eye vision and visual field defects had deteriorated then. The MRI in February 2020 and perimetry in December 2019 are shown in Figs. 1E and F, and 2C, respectively. CAB was resumed at 1.5 mg/week since December 2019.

However, the patient failed to show biochemical and radiological response despite adherence to escalating dosages of CAB after December 2019. Adherence was assessed on the basis of direct questioning of the patient and his next-of-kin as well as counting leftover cabergoline tablets. He reported no side effects with cabergoline. His PRL trend, tumor size, and medications are summarized in Table 1. He was referred to the neurosurgeons in December 2021 for consideration of surgery in view of tumor growth despite high doses of CAB and he finally agreed to surgery in September 2022. During the transsphenoidal surgery, a highly vascular tumor with a firm, adherent capsule was noted. Histology confirmed the presence of a lactotroph macroadenoma that was sparsely granulated and the tumor cells were strongly positive for PIT-1 and PRL. The Ki-67 index was 5% and no mitotic activity was seen. The hyperprolactinemia improved postoperatively from 236.6 ug/L to 100.8 ug/L and he was treated with CAB 2 mg/week.

**Table 1:** Progress of patient between December 2014 and November 2022

Date	PRL (3.4–19.1 ug/L)	FT4 (9.0–19.0 pmol/L)	Total testosterone (4.9–32.0 nmol/L)	Tumor size (cm)	Medication
December 2014	> 470	10.7	5.16	CC: 2.4 TR: 2.0 AP: 2.0	CAB 0.5 mg 2×/week LT4 25 mcg OM
June 2015	121.9	–	–	CC: 1.3 TR: 1.5 AP: 1.8	CAB 0.75 mg 2×/week LT4 75 mcg OM
July 2016	24.1	13.0	4.6	CC: 1.4 TR: 1.7 AP: 1.8	CAB 0.75 mg 2×/week LT4 75 mcg OM
December 2017	> 197.4	12.0	2.2	CC: 1.5 TR: 1.7 AP: 2.1	CAB 0.75 mg 2×/week LT4 75 mcg EOD LT4 100 mcg EOD
February 2020	93.8	10.5	1.4	CC: 2.5 TR: 1.8 AP: 1.7	CAB 0.75 mg 2×/week LT4 50 mcg M-F LT4 25 mcg S,S T 100 mg q28 days
February 2021	160.8	–	–	CC: 2.2 TR: 1.6 AP: 1.7	CAB 0.75 mg 2×/week LT4 25 mcg M-T LT4 50 mcg F-S T 100 mg q28 days
December 2021	271.1	10.2	-	CC: 2.8 TR: 2.0 AP: 1.9	CAB 1.5 mg, 1.5 mg, 1 mg LT4 25 mcg M-T LT4 50 mcg F-S T 100 mg q28 days
April 2022	280.8	-	-	CC: 2.9 TR: 1.9 AP: 1.6	CAB 1.5 mg 3×/week LT4 25 mcg M-T LT4 50 mcg F-S T 100 mg q28 days
September 2022 (preoperative)	236.6	9.3	20.6	CC: 2.7 TR: 1.9 AP: 1.7	CAB 2 mg 3×/week LT4 50 mcg OM T 100 mg q28 days
September 2022 (postoperative)	133.2	–	–	–	CAB 1 mg 2×/week LT4 50 mcg OM T 100 mg q28 days
October 2022	99.9	12.5	–	Intrasellar component: CC: 0.7 TR: 0.9 AP: 1.3 Suprasellar component: CC: 0.9 TR: 2.0 AP: 0.8	CAB 2 mg 3×/week LT4 100 mcg OM T 100 mg q28 days

*AP* anteroposterior, *CAB* cabergoline, *CC* craniocaudal, *EOD* every other day, *F–S* Fridays to Sundays, *FT4* free thyroxine, *LT4* levothyroxine, *M–F* Mondays to Fridays, *M–T* Mondays to Thursday, *OM* once daily, *PRL* prolactin, *S–S* Saturdays to Sundays, *T* testosterone cypionate, *TR* transverse

Six weeks after surgery, he presented with acute deterioration of his vision on the left but MRI showed stable postoperative changes without significant compression on his optic tracts. His hyperprolactinemia had stagnated at 99.9 ug/L, so CAB was increased back to 6 mg/week and levothyroxine was increased in a stepwise manner to 100 mcg daily.

## Discussion

### Results of literature search

Between 1981 and 2022, at least 39 cases of prolactinomas with secondary DA resistance (excluding our case) have been reported in Europe, North and South America, Asia, and Australia. There was adequate information to confirm secondary DA resistance in 18 of these cases. Of the 39 cases, 12 were male, 11 were female, and the gender was not reported in 16 cases. The age of the patients was between 22 and 70 years. A total of 15 cases had macroprolactinomas, 3 had microprolactinomas, and the tumor size was not reported in 21 cases. The time to developing secondary DA resistance was between 10 months and 15 years. Four cases were treated with androgen or estrogen therapy prior to developing secondary DA resistance [7–10]. Importantly, ten cases were ultimately found to have malignant prolactinomas [9, 11–16]. The Ki-67/MIB-1 index was reported in seven cases. Of these, five cases had Ki-67 index > 3% [8, 10, 12, 17, 18], two cases had MIB-1 > 3% [19], and two cases had Ki-67 index < 3% [20, 21]. In eight cases, p53 staining was performed. Of these, three cases were positive [8, 19] and five were negative [17, 18, 20, 21]. We were unable to perform p53 staining in our institute. However, it appears from the available case reports that p53 correlates poorly with the clinical aggressiveness of prolactinomas with secondary DA resistance, while the Ki-67/MIB-1 index appears to be higher in aggressive prolactinomas. The reported cases with secondary DA resistance are summarized in Table 2. The baseline characteristics of these cases are summarized in Table 3.

**Table 2:** Reported cases of prolactinomas with secondary DA resistance

No.	Details at presentation	Progress before secondary DA resistance	Details at secondary DA resistance	Progress after secondary DA resistance	Diagnosis
1 [20]	40, F Symptoms: galactorrhea, secondary amenorrhea Pituitary deficiency: gonadal PRL: 171 ug/L (RI: 6–29.9) Imaging: microadenoma	Treatment: CAB 0.75 mg/week PRL: normalized Imaging: NA	Onset: 3.5 years PRL: 128 ug/L Imaging: macroadenoma	Treatment: CAB 3 mg/week, S PRL: slightly above ULN Imaging: no residual tumor	PRLoma Ki-67: < 5% p53: negative
2 [11]	63, M Symptoms: impotence Pituitary deficiency: gonadal, thyroid, adrenal PRL: 4531 ug/L (RI: NA) Imaging: macroadenoma	Treatment: S, DXT, BRC 7.5 mg/d PRL: normalized Imaging: NA	Onset: 7 years PRL: 1758 ug/L Imaging: slight growth + right frontal metastasis	Treatment: BRC 15 mg/d, Q 600 ug/d, tamoxifen 20 mg/d, S, DXT PRL: 4531 ug/L Imaging: frontal residual metastasis + cyst	Malignant PRLoma
3 [21]	57, F Symptoms: headache, blurred vision, secondary amenorrhea Pituitary deficiency: gonadal, GHD PRL: 6580 ug/L (RI: 3.3–26.6) Imaging: macroadenoma	Treatment: BRC 40 mg/d, CAB 1 mg/week PRL: 59 ug/L (99% reduction) Imaging: NA	Onset: 8 years PRL: 1692 ug/L Imaging: macroadenoma	Treatment: CAB 6 mg/week, S, DXT, CAB 2 mg/week PRL: 118 ug/L (93% reduction) Imaging: stable remnant	PRLoma, no mitosis or cellular atypia Ki-67: < 3% p53: negative
4 [26]	47, M Symptoms: headache, blurred vision, impotence Pituitary deficiency: panhypopituitarism PRL: 6345 ug/L (RI: < 23.5) Imaging: macroadenoma	Treatment: BRC 15 mg/d PRL: 236 ug/L (96% reduction) Imaging: size reduction	Onset: 10 months PRL: 1974 ug/L Imaging: tumor growth	Treatment: BRC 40 mg/d, S, BRC 20 mg/d PRL: 564 ug/L (71% reduction) Imaging: NA	PRLoma
5 [7]	53, M Symptoms: headache, impotence Pituitary deficiency: panhypopituitarism PRL: 1477 ug/L (RI: NA) Imaging: macroadenoma	Treatment: BRC 10 mg/d PRL: normalized Imaging: remnant in left cavernous sinus	Onset: 7 years PRL: NA Imaging: stable remnant	Treatment: BRC 25 mg/d, Q 600 ug/d, CAB 4.5 mg/week PRL: normalized Imaging: stable remnant	PRLoma
6 [12]	63, F Symptoms: diplopia, blurred vision Pituitary deficiency: none PRL: 490 ug/L (RI: < 22) Imaging: macroadenoma	Treatment: BRC 3.75 mg/d PRL: 56 ug/L (89% reduction)	Onset: 1.5 years PRL: 206 ug/L Imaging: tumor growth	Treatment: Q 300 ug/d, CAB (dose: NA), S, DXT, S (spinal metastases), DXT (spinal metastases) PRL: 6000 ug/L Imaging: spinal metastases	Malignant PRLoma, no mitotic activity Ki-67: 10–15%
7 [50]	22, M Symptoms: blurred vision Pituitary deficiency: NA PRL: 1260 ug/L (RI: NA) Imaging: macroadenoma	Treatment: S, BRC 7.5 mg/d PRL: normalized Imaging: NA	Onset: 1 year PRL: 42 ug/L Imaging: macroadenoma	Treatment: S, DXT, BRC 7.5 mg/d PRL: 5.4 ug/L (87% reduction) Imaging: NA	PRLoma
8 [17]	70, M Symptoms: headache Pituitary deficiency: gonadal PRL: 8800 ug/L (RI: 2.5–13) Imaging: macroadenoma	Treatment: CAB 3.5 mg/week PRL: 25 ug/L (99.7% reduction) Imaging: size reduction	Onset: 46 months PRL: 16,000 ug/L Imaging: tumor growth	Treatment: CAB 5 mg/week, S, octreotide 20 mg/month PRL: 16,800 ug/L Imaging: tumor growth	Somatolactotrophoma, no mitotic figures Ki-67: 4% p53: negative
9 [13]	31, F Symptoms: blurred vision, secondary amenorrhea Pituitary deficiency: panhypopituitarism PRL: NA Imaging: macroadenoma	Treatment: S, DXT PRL: 412 ug/L Imaging: stable remnant	Onset: 4 years PRL: 2417 ug/L Imaging: tumor growth	Treatment: S, BRC 20 mg/d, DXT, S (metastasis) PRL: 1604 ug/L Imaging: cerebellar metastasis	Malignant PRLoma, no mitotic activity in pituitary tumor, numerous mitoses in cerebellar metastasis
10 [18]	57, F Symptoms: secondary amenorrhea Pituitary deficiency: gonadal PRL: 75 ug/L (RI: NA) Imaging: microadenoma	Treatment: BRC 5 mg/d, CAB 1 mg/week PRL: normalized Imaging: tumor resolution	Onset: 15 years PRL: 2392 ug/L Imaging: macroadenoma	Treatment: CAB 4 mg/week, S, CAB 1.5 mg/week, DXT PRL: normalized Imaging: stable remnant	PRLoma Ki-67: 11% p53: negative
11 [18]	38, F Symptoms: secondary amenorrhea Pituitary deficiency: gonadal PRL: 124 ug/L (RI: NA) Imaging: microadenoma	Treatment: CAB 1 mg/week PRL: normalized Imaging: NA	Onset: 5 years PRL: 250 ug/L Imaging: macroadenoma	Treatment: CAB 7 mg/week, S, CAB 1.5 mg/week PRL: normalized Imaging: no residual tumor	PRLoma p53: negative
12 [14]	43, M Symptoms: NA Pituitary deficiency: NA PRL: NA Imaging: macroadenoma	Treatment: S, S, S, BRC 55 mg/d PRL: 57 ug/L Imaging: NA	Onset: 4 years PRL: 1000 ug/L Imaging: macroadenoma	Treatment: Q 0.8 mg/d, S, DXT, CAB 14 mg/week, DXT × 4, S × 2, DXT (spinal metastases) PRL: 18,000 ug/L Imaging: no residual tumor, spinal metastases	Malignant PRLoma
13 [15]	40, M Symptoms: headache, blurred vision Pituitary deficiency: panhypopituitarism PRL: > 188 ug/L (RI: < 18.9) Imaging: macroadenoma	Treatment: S, DXT, BRC 40 mg/d PRL: 23.5 ug/L (> 87% reduction) Imaging: no residual tumor	Onset: 5 years PRL: 3807 ug/L Imaging: tumor recurrence	Treatment: S, BRC 40 mg/d, S, chemotherapy, S, chemotherapy PRL: 23,500 ug/L Imaging: tumor growth, left vertebral artery metastasis	Malignant PRLoma
14 [8]	62, M Symptoms: visual loss, weight loss, loss of pubic hair Pituitary deficiency: panhypopituitarism PRL: 2,598,489 ug/L (RI: 2115–17,625) Imaging: macroadenoma	Treatment: S, CAB 3 mg/week PRL: normalized Imaging: 0.5 cm remnant	Onset: 5 years PRL: 143,350 ug/L Imaging: macroadenoma	Treatment: CAB 7 mg/week, S, S, DXT PRL: 5,041,643 ug/L Imaging: tumor growth	PRLoma, rare mitotic figures in first two resections, 9–10 mitoses/10 HPF in third resection Ki-67: 3% in first two resections, 20–30% in third resection p53: negative in first two resections, positive in third resection
15 [9]	42, F Symptoms: headache, blurred vision Pituitary deficiency: panhypopituitarism PRL: 728 ug/L (RI: < 23) Imaging: macroadenoma	Treatment: DXT, S, S, S, BRC 15 mg/d PRL: 43 ug/L (94% reduction) Imaging: NA	Onset: 2 years PRL: 313 ug/L Imaging: macroadenoma	Treatment: BRC 30 mg/d, DXT, BRC 37.5 mg/d, DXT, P 25 ug/d PRL: 12,800 ug/L Imaging: scalp metastases	Malignant PRLoma
16 [19]	45, M Symptoms: NA Pituitary deficiency: NA PRL: 3000 ug/L (RI: NA) Imaging: macroadenoma	Treatment: CAB 1 mg/week PRL: 54 ug/L (98% reduction) Imaging: NA	Onset: 9 years PRL: 1519 ug/L Imaging: macroadenoma	Treatment: S PRL: NA Imaging: size reduction	PRLoma MIB-1: > 3% p53: positive
17 [19]	58, F Symptoms: NA Pituitary deficiency: NA PRL: 240 ug/L (RI: NA) Imaging: macroadenoma	Treatment: BRC 15 mg/d, CAB 9 mg/week PRL: 29 ug/L (88% reduction) Imaging: NA	Onset: 6 years PRL: 909 ug/L Imaging: macroadenoma	Treatment: S PRL: NA Imaging: size reduction	PRLoma MIB-1: > 3% p53: positive
18 [10]	55, M Symptoms: diplopia, visual loss, impotence Pituitary deficiency: gonadal, thyroid PRL: 364 ug/L (RI: 2.8–17.9) Imaging: macroadenoma	Treatment: BRC 15 mg/d PRL: normalized Imaging: residual tumor	Onset: 5 years PRL: 229 ug/L Imaging: macroadenoma	Treatment: CAB 5 mg/week, S, CAB 14 mg/week + BRC 10 mg/d, S, S, DXT PRL: 470 ug/L Imaging: residual tumor	PRLoma Ki-67: 10–15% in first resection, 20–25% in second resection, > 25% in third resection
19–24 [51]	Brue *et al*. described six patients with PRLomas with secondary DA resistance. All patients had normalized PRL within 1 year of treatment with subsequent increase in PRL. The onset of secondary DA resistance was 2–6 years. All patients were treated with BRC and none were treated with CAB or S. No further details were provided				
25 [52]	Laboy-Ortiz *et al*. described at 42-year-old male with PRLoma who developed secondary DA resistance after 13 months. No further details were provided				
26–29 [16]	Pernicone *et al*. described four patients with malignant PRLomas with secondary DA resistance. All patients had early response to DA followed by escape from control. No further details were provided				
30–31 [53]	Astaf’eva *et al*. described two patients with PRLomas with secondary DA resistance. One patient achieved disease control after DXT. No further details were provided				
32–39 [24]	Vroonen *et al*. described eight patients with late resistance to DA but did not provide details on these cases				

*BRC* bromocriptine, *CAB* cabergoline, *DA* dopamine agonists, *DXT* radiotherapy, *F* female, *GHD* growth hormone deficiency, *HPF* high power field, *M* male, *MRI* magnetic resonance imaging, *NA* not available, *P* pergolide, *PRL* prolactin, *PRLoma* prolactinoma, *Q* quinagolide, *RI* reference interval, *S* surgery, *ULN* upper limit of normal. DA doses shown are the maximal tolerated/effective dose at each phase of follow-up, onset of secondary DA resistance are stated as years from first presentation, PRL expressed in mU/L were converted to ug/L by dividing the values by 21.2

**Table 3:** Clinical features of prolactinoma patients with secondary DA resistance between 1981 and 2016

Characteristic	Value
Gender, male	12
Gender, female	11
Gender, unknown	8
Age (range), years	22–70
Androgen/estrogen replacement therapy	4
Macroadenoma	15
Microadenoma	3
Tumor size not reported	13
Time to secondary resistance (range), years	0.8–15
Malignant prolactinomas	10

### Definition and frequency of DA resistance

There is no standardized definition for DA resistance. The most commonly used definition is failure to normalize PRL and/or reduce tumor volume by at least 50% using conventionally maximum or maximally tolerated doses of DA (for example, BRC 7.5 mg/day or CAB 2 mg/week) [5, 6, 22–24]. Doses of CAB up to 12 mg/week and BRC up to 30 mg/day were required in rare cases to achieve control of prolactinomas [25]. Ono *et al*. also recommend using DA at the maximum tolerated dose for at least 12 months before concluding that DA resistance is present [25].

There is also no proposed definition for secondary DA resistance. This makes it difficult to ascertain the frequency of secondary DA resistance. Furthermore, a significant proportion of cases of secondary DA resistance were reported before CAB was commercially available in 1992, so some of these may not be true cases of DA resistance. The majority of the reported cases of secondary DA resistance had achieved normoprolactinemia before PRL levels started to rise. Ten of these cases did not achieve normoprolactinemia, but most of them had reduction of PRL by more than 80% from baseline [9, 12, 15, 17, 19, 21, 26], with the exception of two cases where PRL fell by 48% [13] and 73% [14]. The PRL level in our patient had fallen by 84% from baseline in the initial 26 months of treatment before he developed secondary DA resistance. Thereafter, his PRL level stagnated between 144.4 ug/Land 282.5 ug/L despite escalating CAB from 1.5 mg/week to 6 mg/week. Given his remarkable initial response to CAB, we believe there should be no debate on the development of secondary DA resistance in this patient.

### Differential diagnoses of secondary DA resistance

Before concluding that a patient has secondary DA resistance, it is pertinent to exclude non-adherence to therapy. There is also a need to consider if the patient has a non-lactotroph pituitary adenoma with stalk effect. Stalk effect can sometimes happen when the tumor comprises two populations of lactotroph and non-lactotroph cells; the non-lactotroph component causes the stalk effect while the lactotroph component caused the initial improvement with DA [17]. Occasionally, the tumor may expand due to hemorrhage, exerting a new stalk effect after an initial response. Certain medications such as spiramycin [27] may accelerate BRC clearance and reduce its efficacy. Gonadal steroid replacement may induce DA resistance in lactotrophs, and this was seen in four cases with secondary DA resistance [7–10]. An important differential diagnosis is malignant prolactinoma, which was reported in at least 10 of the 39 cases of secondary DA resistance [9, 11–16]. The PRL levels in such cases tended to be markedly elevated despite resolution or stability of the primary pituitary lesions, prompting exploration of additional foci of PRL secretion that led to discovery of metastases. Other differential diagnoses include pregnancy, physiologic causes of hyperprolactinaemia (for example, stress), interim development of pathologic causes of hyperprolactinaemia (for example, renal or hepatic impairment), and introduction of new medications (for example, antipsychotics).

### Mechanisms of secondary DA resistance

DA bind to dopamine type 2 receptors (D2R) in prolactinomas resulting in inhibition of PRL secretion as well as reduction of tumor size [28]. True DA resistance has been attributed to various D2R defects in prolactinomas. Breidahl *et al*. demonstrated that secondary DA resistance in their patient developed due to growth of a population of lactotrophs that had reduced D2R expression [26]. Some lactotrophs may also produce structurally abnormal D2R that have reduced DA binding capacity [19, 29, 30]. The loss of D2R expression or affinity to DA may arise from late mutations in tumor cells [20], explaining the secondary resistance. D2R is expressed as short and long isoforms in human beings. The short isoform induces intracellular signalling via the MAPK and PI3k pathways while the long isoform inhibits these pathways [31]. A lower ratio of the short-to-long isoform has been shown to reduce the response of lactotrophs to DA [19, 30, 32, 33]. We were unable to perform D2R staining in our institute and were thus unable to ascertain if the secondary DA resistance in this patient was due to reduced D2R density.

It has been postulated that angiogenic factors (for example, vascular endothelial growth factor) promote tumor cell proliferation and angiogenesis in prolactinomas resistant to DA [17, 34]. This may confer the aggressive and invasive behavior of such tumors. The prolactinoma in our patient was noted to be highly vascular. However, we were unable to test for the presence of angiogenic factors in our institute.

Secondary DA resistance was previously reported in four cases that received gonadal steroid replacement [7–10]. Similarly, our patient started exhibiting secondary DA resistance after he was started on testosterone replacement therapy. This can probably be explained by the aromatization of testosterone to estrogen, which then regulates PRL gene expression, downregulates D2R, and stimulates lactotroph hyperplasia [35, 36]. However, in the prospective study conducted by Auriemma *et al*., DA resistance was not seen in nine hypogonadal males with prolactinomas who were started on testosterone replacement therapy. These patients had improvements in their metabolic profiles instead [37]. Molitch *et al*. [6] recommended the addition of aromatase inhibitors to counteract the estrogenic effect from testosterone therapy on the basis of a case report by Gillam *et al*. [38].

DA resistance has also been attributed to cell signalling defects. For example, defects in nerve growth factor (NGF) can disrupt autocrine growth factor signalling pathways in lactotrophs and disinhibit cell proliferation, causing prolactinomas to develop DA resistance [39]. Response to DA was restored after prolactinoma cells were exposed to NGF [40] in cell culture studies. Filamin-A is a cytoskeleton protein that is involved in D2R expression and function. D2R cannot be trafficked to the cell membrane in the absence of filamin-A [41]. Prolactinomas with DA resistance have reduced filamin-A expression, and inhibition of filamin-A expression using small interfering ribonucleic acids led to DA resistance [42]. In addition, micro ribonucleic acids may interfere with intracellular signalling and induce fibrosis in DA-resistant prolactinomas [43], resulting in failure of tumor volume reduction [17].

### Treatment strategies in secondary DA resistance

In patients who developed resistance to BRC, switching to CAB is a reasonable first-line strategy. This is because CAB has superior efficacy in normalizing PRL levels and reducing tumor size due to its higher affinity for D2R, leading to better cytocidal effects [44–46]. About 33% of patients with macroprolactinomas fail to achieve tumor shrinkage by at least 50% using BRC. Of these, 70% respond to CAB [6]. CAB also has superior side effect profile compared with other DA [45, 47, 48].

In patients with concurrent hypopituitarism or central hypothyroidism, we recommend thyroid replacement therapy to keep the serum free thyroxine in the upper half of the reference interval. As thyrotropin releasing hormone stimulates prolactin release [49], the negative feedback exerted by thyroxine on hypothalamic thyrotropin releasing hormone secretion can help to reduce the stimulation of prolactin release.

Our patient was recommended for surgical resection after high doses of CAB failed to normalize his PRL and reduce tumor volume. Surgical resection was also performed in 17 of the reported cases. Three of the reported cases with secondary DA resistance received DA therapy in conjunction with surgery and achieved reduction in PRL by more than 50% after surgery [18, 20, 26]. A total of 11 cases had surgery and required extra treatment modalities (for example, radiotherapy) in addition to DA therapy. Of these, three achieved reductions in PRL by more than 50% [18, 21, 50]. Thus, it appears that response to surgical resection is variable. Surgical resection has the additional advantage of providing tissue samples for immunohistological evaluation for cellular atypia, mitotic burden, and proliferation index. None of the cases treated with somatostatin analogue [17], tamoxifen [11], and chemotherapy [15] achieved significant reduction in PRL levels. Temozolomide was proposed as a plausible option by Sbardella [8] *et al*. We recommend a low threshold for repeating MRI in patients whose histopathological samples showed high proliferative indices (for example, Ki-67 index > 3%), and who have unexpected or unexplained rise in PRL from their postoperative baseline, as this group of patients appear to be at higher risk for local recurrence and metastases. We also recommend further imaging to detect metastases if the MRI were to show stable changes in the parasellar region in this group of patients.

## Conclusion

Secondary DA resistance in prolactinomas is a rare phenomenon and has been reported to occur up to 15 years after presentation. Mixed tumors, D2R defects, and abnormal cell signalling pathways may explain the development of secondary DA resistance. Importantly, malignant prolactinomas need to be considered as a differential diagnosis in such cases. Early surgery for tumor resection or debulking may confer additional biochemical control and provide tissue for immunohistological evaluation.

## Data Availability

The datasets generated and/or analyzed during the current study are available from the corresponding author on reasonable request.

## Abbreviations

AP: Anteroposterior
BRC: Bromocriptine
CAB: Cabergoline
CC: Craniocaudal
DA: Dopamine agonists
D2R: Dopamine type 2 receptors
DXT: Radiotherapy
EOD: Every other day
F: Female
F–S: Fridays to Sundays
FT4: Free thyroxine
GHD: Growth hormone deficiency
HPF: High power field
LT4: Levothyroxine
M: Male
M–F: Mondays to Fridays
MRI: Magnetic resonance imaging
M–T: Mondays to Thursday
NA: Not available
NGF: Nerve growth factor
OM: Once daily
P: Pergolide
PRL: Prolactin
PRLoma: Prolactinoma
Q: Quinagolide
RI: Reference interval
S: Surgery
S–S: Saturdays to Sundays
T: Testosterone cypionate
TR: Transverse
ULN: Upper limit of normal
